# Proteomic profiling of *FBXW7*‐mutant serous endometrial cancer cells reveals upregulation of PADI2, a potential therapeutic target

**DOI:** 10.1002/cam4.3013

**Published:** 2020-04-05

**Authors:** Mary Ellen Urick, Daphne W. Bell

**Affiliations:** ^1^ Cancer Genetics and Comparative Genomics Branch National Human Genome Research Institute National Institutes of Health Bethesda MD USA

**Keywords:** FBXW7, mutation, PADI2, proteomics, UCHL1, uterine neoplasms

## Abstract

**Background:**

Despite advancements over the past decade revealing molecular aberrations characteristic of endometrial cancer (EC) subtypes, serous ECs remain difficult to treat and associated with poor outcomes. This is due, in part, to the rarity of these tumors within clinical trials and the inability to directly target the most frequent genomic abnormalities. One of the most commonly somatically mutated genes in serous ECs is the tumor suppressor *F‐box and WD repeat domain containing 7* (*FBXW7*).

**Methods:**

To identify changes in protein expression associated with *FBXW7* mutation, we clustered regularly interspaced short palindromic repeats (CRISPR)‐edited ARK4 *FBXW7* nonmutant serous EC cells to insert recurrent *FBXW7* mutations. We then compared the liquid chromatography tandem mass spectrometry‐based proteomic profiles of CRISPR‐edited ARK1 and ARK4 serous EC cells to matched parental cells.

**Results:**

Among distinct total and phosphorylated proteins that were significantly differentially expressed in *FBXW7*‐mutant cell lines compared to matched parental lines, we identified increased PADI2 (peptidyl arginine deiminase 2) expression in all ARK1 and ARK4 CRISPR‐edited *FBXW7*‐mutant cell lines. We further confirmed the correlation between *FBXW7* mutation and increased PADI2 expression in a third biological background, JHUEM‐1 endometrioid EC cells. Finally, we established that PADI2 protein is expressed in primary serous endometrial tumors.

**Conclusion:**

Our findings provide novel insight into proteomic changes associated with *FBXW7* mutation in serous ECs and identify PADI2 as a novel potential therapeutic target for these tumors.

## Graphical abstract

Mass spectrometry‐based profiling identified proteomic changes associated with recurrent somatic *FBXW7* mutations and shared in two biologically distinct serous endometrial cancer cell lines. Most notably, a correlation between PADI2 protein and *FBXW7* mutation was demonstrated in serous endometrial cancer cells and confirmed in an endometrioid endometrial cancer cell line. PADI2 protein expression was further demonstrated in primary serous endometrial tumors.

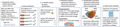

## 1 INTRODUCTION

Although the most common histotype of endometrial cancers (ECs), endometrioid EC, can frequently be effectively treated through hysterectomy, serous EC is a rarer subtype that is often associated with metastasis, recurrence, therapy resistance, and poor outcome.^1^, ^2^ Serous ECs and other clinically aggressive subtypes exhibit frequent somatic mutation of the tumor suppressor *FBXW7* (*F‐box and WD repeat domain containing 7*; also known as *FBW7*, *CDC4*, and *hAGO*). Somatic *FBXW7* mutations occur in 15%‐29% of serous ECs, 11%‐39% of uterine carcinosarcomas, 13%‐25% of clear cell ECs, and 0%‐15% of endometrioid ECs (reviewed in Ref. [^3^]). In serous ECs, somatic mutation hotspots occur at *FBXW7* codons 423, 465, 479, and 505.^4^, ^5^, ^6^

Research on serous ECs is hindered in part due to the rarity of these tumors and availability of only small numbers of cell lines. An ideal model system to examine the effects of *FBXW7* mutation has been developed through CRISPR editing of ARK1 serous EC cells to insert recurrent *FBXW7* somatic mutations.^7^ Research comparing the levels of a small number of proteins in parental and CRISPR‐edited ARK1 cell lines provided the first insights into the direct biochemical effects of *FBXW7* mutations in the context of serous EC: increased phosphorylation of seven cancer‐related proteins detected by Western blot.^7^ Equivalent protein changes also occurred in ARK1 and ARK2 cells transiently expressing mutant *FBXW7*.^7^

Here, we CRISPR‐edited ARK4 serous EC cells to insert recurrent *FBXW7* somatic mutations and performed large‐scale tandem mass spectrometry‐based proteomic profiling on both ARK1 and ARK4 parental and derivative cells. Our findings provide novel insight into the proteomic changes associated with recurrent *FBXW7* mutation in two biologically distinct serous EC cell lines, which include new potential therapeutic targets, most notably PADI2 (peptidyl arginine deiminase 2). We orthogonally validated increased PADI2 protein expression in ARK1 and ARK4 *FBXW7*‐mutant cells via Western blot and demonstrated PADI2 protein expression in primary serous endometrial tumors. To further solidify the correlation of increased PADI2 expression to *FBXW7* mutation, we used CRISPR editing to revert the endogenous *FBXW7* c.C1513T (p.R505C) mutation in JHUEM‐1 endometrioid EC cells to a wild‐type genotype and showed that PADI2 expression was decreased in CRISPR‐edited *FBXW7* nonmutant JHUEM‐1 cells compared to parental cells.

## 2 MATERIALS AND METHODS

**Figure 1:**
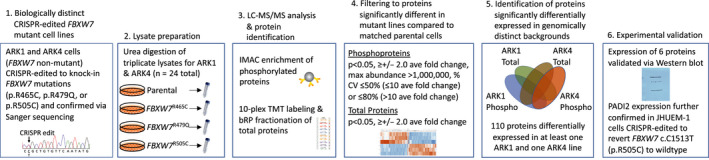
Outline of experimental procedures for proteomic analysis of CRISPR‐edited *FBXW7*‐mutated serous endometrial cancer cell lines. Ave, average; CV, coefficient of variance; IMAC, immobilized metal affinity chromatography; LC‐MS/MS, liquid chromatography tandem mass spectrometry; max, maximum; TMT, Tandem Mass Tags

A summary of methods utilized in this manuscript is provided in Figure 1. The research conducted in this study was excluded from IRB Review per 45 CFR 46 and NIH policy for the use of specimens/data.

### 2.1 Cell culture

ARK1 and ARK4 serous EC cell lines were established^8^ and kindly provided by Dr Alessandro A. Santin (Yale University School of Medicine, New Haven, CT). Both lines were maintained in RPMI + 10% fetal bovine serum (FBS) at 37°C in a humidified atmosphere with 5% CO_2_. JHUEM‐1 endometrioid EC cells were purchased from Riken Bioresource Research Center and maintained in DMEM/HamF12 + 15% FBS at 37°C in a humidified atmosphere with 5% CO_2_. Cells were counted with a Countess Cell Counter (Thermo Fisher Scientific). All cell lines were authenticated by Laragen, Inc using short tandem repeat profiling prior to shipment to the Washington University Genome Engineering and iPSC Center (GEIC). All cell lines were verified mycoplasma‐free by GEIC and authenticated again at the time frozen stocks were established.

### 2.2 Generation of CRISPR‐edited *FBXW7*‐mutated cell lines

ARK4 cells were CRISPR‐edited by GEIC to incorporate *FBXW7* c.C1393T (p.R465C), c.G1436A (p.R479Q), and c.C1513T (p.R505C). ARK4 was edited following published methods^7^ with one exception: RNP complexes were assembled by combining 200 pmol of Alt‐R gRNA (Integrated DNA Technologies) with 80 pmol of Cas9 protein (California Institute for Quantitative Biosciences) at room temperature for 10 minutes. JHUEM‐1 cells were CRISPR‐edited by GEIC to remove the endogenous *FBXW7* c.C1513T (p.R505C) mutation following the methods used for ARK4.

ARK1 and ARK4 parental cells lack *FBXW7* exonic mutations (verified by Sanger sequencing as described below); ARK1 exhibits *FBXW7* copy number loss, and ARK4 has an unknown *FBXW7* copy number status.^9^ JHUEM‐1 parental cells harbor *FBXW7* c.C1513T (p.R505C) and exhibit diploid copy number status.^10^

### 2.3 Copy number analysis

Because the JHUEM‐1 CRISPR‐edited clone did not harbor silent blocking modifications intended to prevent recutting during CRISPR modification, GEIC completed copy number analysis using the Hs02590357_cn TaqMan^TM^ Copy Number Assay (Thermo Fisher Scientific) according to the manufacturer's protocol. Assay controls were the human RNase P TaqMan^TM^ Copy Number Assay (4403328, Thermo Fisher Scientific) and GEIC’s AN1 female‐induced pluripotent stem cells. DNA was extracted using the DNeasy Blood & Tissue kit (Qiagen) following the manufacturer's protocol and data were analyzed using CopyCaller Software (Thermo Fisher Scientific). Results showed matching diploid status of JHUEM‐1 parental cells and the CRISPR‐edited derivative line (data not shown).

### 2.4 DNA extraction, polymerase chain reaction (PCR) amplification, and Sanger sequencing

DNA was extracted using the Gentra^®^ Puregene^®^ Kit (Qiagen) according to the manufacturer's instructions. *FBXW7* primer sequences are available upon request. All coding exons of *FBXW7* were sequenced in ARK4 and JHUEM‐1 parental and derivative lines to verify CRISPR edits (Figure S1) using PCR conditions, purification, and Sanger sequencing exactly as previously reported.^7^

### 2.5 Lysate preparation and liquid chromatography tandem mass spectrometry (LC‐MS/MS) analysis

Cells were plated into four 150 mm dishes at a density of 4 × 10^6^ cells per dish and scraped into a total of 4 mL urea lysis buffer (Cell Signaling Technology (CST)^®^) the following day. Triplicate lysates were prepared for each cell line and were immediately frozen on dry ice before shipment to CST^®^. Cellular extracts were sonicated, centrifuged, reduced with dithiothreitol, and alkylated with iodoacetamide. For phosphorylated protein analysis, 500 μg total protein was digested with trypsin, purified over C18 columns (Waters), and used for immobilized metal affinity chromatography (IMAC) enrichment with Fe‐NTA magnetic beads (CST^®^) as previously described.^11^ For total protein analysis, 100 μg of each sample was digested with LysC/trypsin, labeled with TMT10plex^TM^ reagent (Thermo Fisher Scientific), bRP fractionated (96 fractions concatenated nonsequentially to 12), and C18 purified as previously described.^12^ LC‐MS/MS analysis was performed using an Orbitrap‐Fusion^TM^ Lumos^TM^ Tribrid^TM^ mass spectrometer (Thermo Fisher Scientific) as previously described^11^, ^12^.

For IMAC phosphopeptide analysis, replicate injections were used for each sample and an HCD‐MS2 acquisition method was used with the following parameters: method duration 181 minutes, user‐defined lock mass 371.10123, Orbitrap resolution 120K, scan range 300‐1500 m/z, maximum injection time 50 ms, AGC target 4E5, dynamic exclusion = 1, exclusion duration 30 seconds, mass tolerance ∓ 10 ppm, max intensity 1E20, min intensity 500, MS2 isolation mode quadrupole, MS2 isolation window 1.6, activation type HCD, collision energy mode fixed, collision energy 27, detector type Orbitrap, Orbitrap resolution 30K, max injection time 54 ms, and AGC target 5E4.

For total proteome analysis, an MS3 acquisition method was used to reduce ion interference and ratio compression with the following parameters: method duration 210 minutes, user‐defined lock mass 371.10123, Orbitrap resolution 120K, scan range 350‐1400 m/z, maximum injection time 100 ms, AGC target 5E5, dynamic exclusion = 1, exclusion duration 120 seconds, mass tolerance ∓ 7 ppm, MS2 isolation mode quadrupole, MS2 isolation window 0.4, activation type CID, collision energy mode fixed, collision energy 35, detector type IonTrap, max injection time 10 ms, AGC target 2E4, MS3 isolation mode quadrupole, isolation window 0.7, multi‐notch isolation, MS2 isolation window 3 m/z, number of notches = 10, collision energy mode fixed, collision energy 65, detector type Orbitrap, Orbitrap resolution 50K, max injection time 150 ms, and AGC target 2.5E5.

### 2.6 Peptide and protein identification

Mass spectra were evaluated by CST^®^ using SEQUEST and the GFY‐Core platform (Harvard University).^13^, ^14^, ^15^ Searches were performed against the 20180718 update of the Uniprot *Homo sapiens* database with a mass accuracy of ± 50 ppm for precursor ions and 0.02 Da for product ions. Total proteome data were filtered to a 1% peptide‐level false discovery rate (FDR) with mass accuracy ± 5 ppm on precursor ions and presence ions; IMAC data were filtered to samples with a phosphorylated residue prior to filtering to a 1% protein‐level FDR. All IMAC quantitative results were generated using Skyline^16^ to extract the integrated peak area of the corresponding peptide assignments. Accuracy of quantitative data was ensured by manual review in Skyline or in the ion chromatogram files. TMT quantitative results were generated using the GFY‐Core platform (Harvard University).

### 2.7 Filtering of proteins significantly differentially expressed in *FBXW7*‐mutation knock‐in cells

Total proteins were filtered to those significantly (*P* < .05) differentially expressed in *FBXW7‐*mutation knock‐in cells compared to matched parental cells with ≥±2.0 average fold change. *P*‐values were based on a two‐tailed *t* test using the signal:noise for each mutation knock‐in cell line versus the matched parental cell line across a minimum of three replicates. Phosphorylated peptides were filtered using these same criteria with additional filters of maximum abundance > 1 000 000 and percent coefficient of variance (%CV) ≤50% for peptides with ≤ 10 average fold change or ≤ 80% for peptides with > 10 average fold change. Unsupervised hierarchical clustering was performed using Partek^®^ Genomics Suite^®^.

### 2.8 Functional annotation of proteins significantly differentially expressed in *FBXW7*‐mutation knock‐in cells

Proteins were functionally annotated using g:Profiler^17^ and Ingenuity Pathway Analysis (IPA) (QIAGEN Inc, https://www.qiagenbioinformatics.com/products/ingenuitypathway‐analysis).

### 2.9 Western blot analysis

Protein isolation, quantification, gel electrophoresis, and transfer were performed following published methods^7^ 1 day after plating 5.5 × 10^5^ cells per 60‐mm dish. Protein was extracted from six primary serous endometrial tumors using a protocol modified from Peña‐Llopis and Brugarolas.^18^ Briefly, tissue was homogenized in 10 volumes of RIPA buffer (Thermo Fisher Scientific) containing complete tablet protease inhibitors (Sigma‐Aldrich), 0.1‐mmol/L NaVO_4_, and 10m‐mol/L NaF (New England BioLabs) while alternating between wet and dry ice. Lysates were centrifuged twice through a QIAshredder column (Qiagen), then cleared using centrifugation.

Proteins were subjected to electrophoresis into Bolt 8% or 10% Bis‐Tris Gels (Thermo Fisher Scientific) and wet transferred to PVDF membranes (Bio‐Rad Laboratories). Blots were blocked in Tris‐buffered saline containing 1% Tween 20 (TBST) + 5% milk and incubated in the following antibodies from CST^®^ according to their suggested protocols: PADI2 (#97647), UCHL1 (#13179), P‐MARCKS (#8722), MARCKS (#5607), NDRG1 (#9485), TGM2 (#3557), and EPLIN (#50311). As a loading control, blots were incubated in β‐Actin antibody (A2228; Sigma‐Aldrich) overnight at 4°C at a dilution of 1:5000 in TBST + 5% milk. Secondary antibodies were Cell Signaling # 7074 and 7076. Proteins were detected using Clarity™ Western ECL Substrate (Bio‐Rad Laboratories) prior to film exposure (Carestream Health, Inc or Agfa Healthcare NV) and development using a film processor (Konica Minolta, Ramsey, NJ). To enable probing with multiple antibodies, blots were incubated in Restore™ Western Blot Stripping Buffer (Thermo Fisher Scientific). All Western blots are representative of a minimum of three lysate replicates.

## 3 RESULTS

We CRISPR‐edited the ARK4 serous EC cell line to generate three derivative cell lines, each harboring one recurrent *FBXW7* mutation (p.R465C, p.R479Q, or p.R505C) (Figure S1). To identify proteomic changes associated with recurrent *FBXW7* mutations in serous ECs, we performed LC‐MS/MS proteomic profiling for ARK1^7^ and ARK4 parental and CRISPR‐edited *FBXW7*‐mutant cell lines in triplicate and compared phosphorylated and total protein expression of *FBXW7*‐mutated cell lines to that of the matched *FBXW7* nonmutated parental lines.

**Figure 2:**
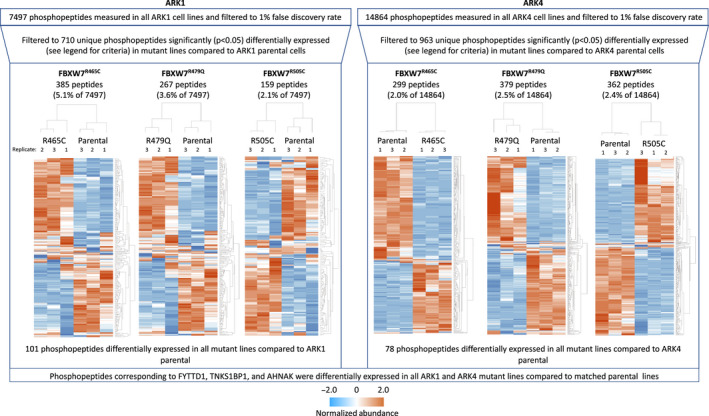
Unsupervised hierarchical clustering of normalized abundance of filtered phosphorylated peptides significantly (*P* < .05) differently expressed in CRISPR‐edited *FBXW7*‐mutated cells compared to respective *FBXW7* nonmutant parental cells in replicate lysates prepared for each cell line. Phosphopeptides shown met the following filtering criteria: ≥±2.0 average fold change, maximum abundance > 1 000 000, and coefficient of variance (CV) ≤50% for peptides w/ ≤10 average fold change or CV ≤ 80% for peptides w/ >10 average fold change. Fold change is not shown on this figure and was calculated within replicates, comparing mutant line expression to parental. Protein names and fold change values are provided in Figures 3, 4 and Tables S1‐S6

**Figure 3:**
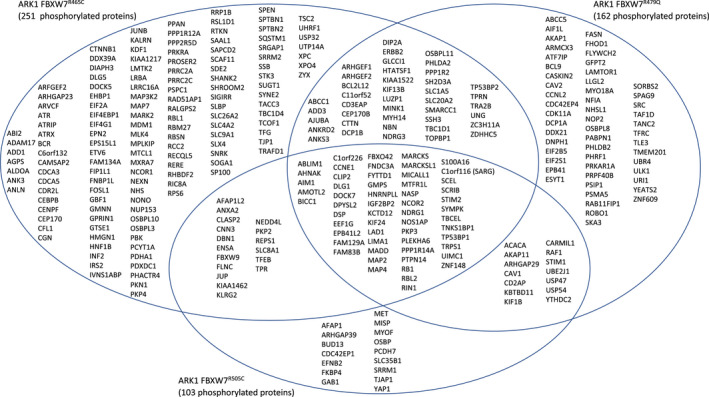
Venn diagram of phosphorylated proteins significantly (*P* < .05) differently expressed in CRISPR‐edited *FBXW7*‐mutated ARK1 cells compared to *FBXW7* nonmutant ARK1 parental cells. Proteins shown met the following filtering criteria: ≥±2.0 average fold change compared to parental ARK1, maximum abundance > 1 000 000, and coefficient of variance (CV) ≤50% for peptides w/ ≤10 average fold change or CV ≤ 80% for peptides w/ >10 average fold change. Note, this figure shows proteins while Figure 2 depicts peptides (multiple peptides can map to the same protein)

**Figure 4:**
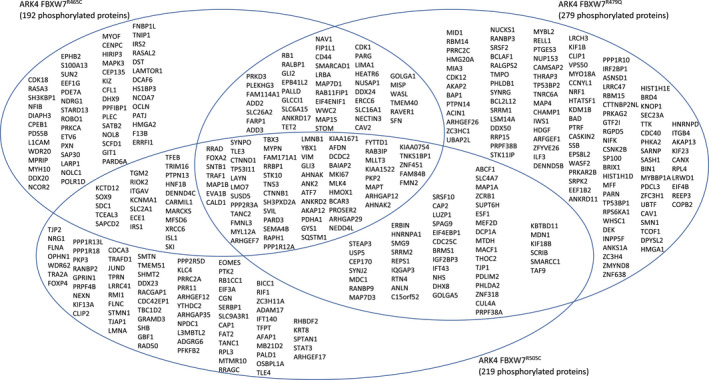
Venn diagram of phosphorylated proteins significantly (*P* < .05) differently expressed in CRISPR‐edited *FBXW7*‐mutated ARK4 cells compared to *FBXW7* nonmutant ARK4 parental cells. Proteins shown met the following filtering criteria: ≥±2.0 average fold change compared to parental ARK4, maximum abundance > 1 000 000, and coefficient of variance (CV) ≤50% for peptides w/ ≤10 average fold change or CV ≤ 80% for peptides w/ >10 average fold change. Note, this figure shows proteins while Figure 2 depicts peptides (multiple peptides can map to the same protein)

Following stringent data filtration (see Methods and Figure 1), 710 and 963 phosphorylated peptides were significantly differentially expressed in ARK1 and ARK4 *FBXW7*‐mutated cell lines, respectively, compared to matched parental lines (Figures 2, 3, 4, Tables S1‐S6). Phosphopeptides corresponding to 127 proteins were significantly differentially expressed in at least two *FBXW7*‐mutant ARK1 cells compared to ARK1 parental cells (Figure 3) and mapped most significantly (*P* < 2.7 × 10^−6^) to the Gene Ontology (GO) terms *cytoskeletal binding*, *cadherin binding*, and *cell adhesion molecule binding*; the only significant (*P* < 8.5 × 10^−5^) KEGG pathway was *tight junction* (Table S7). Phosphopeptides corresponding to 185 proteins were significantly differentially expressed in at least two *FBXW7*‐mutant ARK4 cell lines compared to ARK4 parental cells (Figure 4) and mapped most significantly (*P* < 2.7 × 10^−10^) to the GO terms *protein binding*, *cytoskeletal protein binding*, and *actin binding*; the only significant (*P* < 2.3 × 10^−6^) KEGG pathway was *adherens junction* (Table S8). Phosphopeptides corresponding to three proteins (FYTTD1 (forty‐two‐three‐domain containing 1), TNKS1BP1 (tankyrase 1 binding protein 1), and AHNAK (AHNAK nucleoprotein)) were significantly differentially expressed in all ARK1 and ARK4 *FBXW7*‐mutated cell lines.

**Figure 5:**
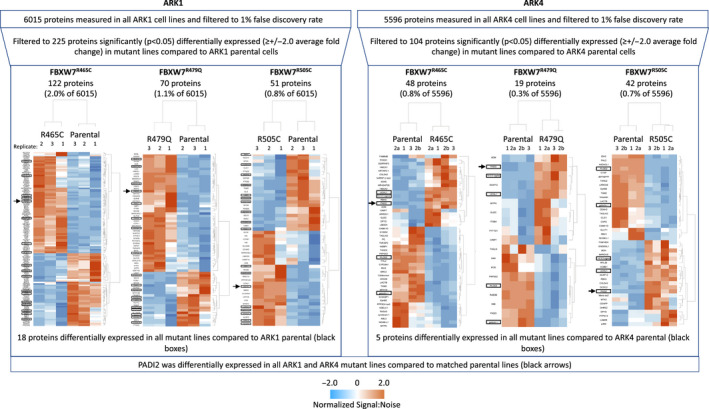
Unsupervised hierarchical clustering of normalized signal:noise for total proteome peptides significantly (*P* < .05) differently expressed in CRISPR‐edited *FBXW7*‐mutated cells compared to respective *FBXW7* nonmutant parental cells in replicate lysates prepared for each cell line. ARK4 replicate 2 was run twice (to fill extra 10‐plex runs; both values for replicate 2 were used in final calculations). Fold change is not shown on this figure and was calculated within replicates, comparing mutant line expression to parental. Proteins significantly differentially expressed in all three ARK1 or ARK4 *FBXW7*‐mutant lines compared to respective parental lines are boxed. Protein names and fold change values are provided in Figure 6 and Tables S9‐S14. Arrows indicate PADI2, which was significantly differentially expressed in all three ARK1 and ARK4 *FBXW7*‐mutant lines compared to respective parental controls

**Figure 6:**
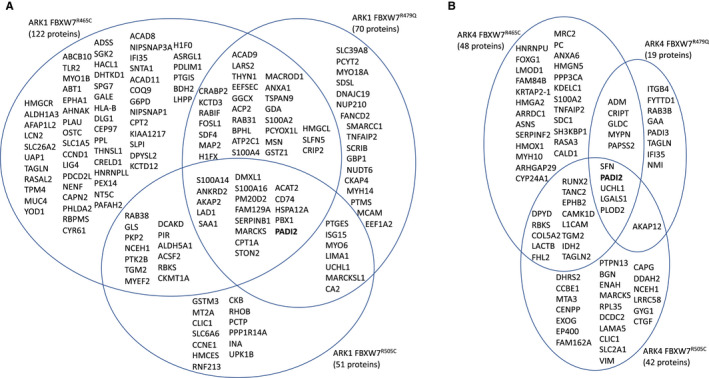
Venn diagrams of total proteome proteins significantly (*P* < .05) differently expressed (≥±2.0 average fold change) in (A) CRISPR‐edited ARK1 *FBXW7*‐mutant cell lines  and (B) CRISPR‐edited ARK4 *FBXW7*‐mutant cell lines compared to matched parental lines

In total proteome analyses, 225 and 104 proteins were significantly differentially expressed among ARK1 and ARK4 *FBXW7*‐mutated cell lines, respectively, compared to matched parental lines (Figures 5, 6, Tables S9‐14). Sixty‐six proteins were significantly differentially expressed in at least two ARK1 *FBXW7*‐mutant lines (Figure 6A) and significantly (*P* < .002) mapped to the *extracellular exosome* GO term, while 24 proteins were significantly differentially expressed in at least two ARK4 *FBXW7*‐mutant lines, compared to parental lines (Figure 6B, Tables S15‐16).

**Figure 7:**
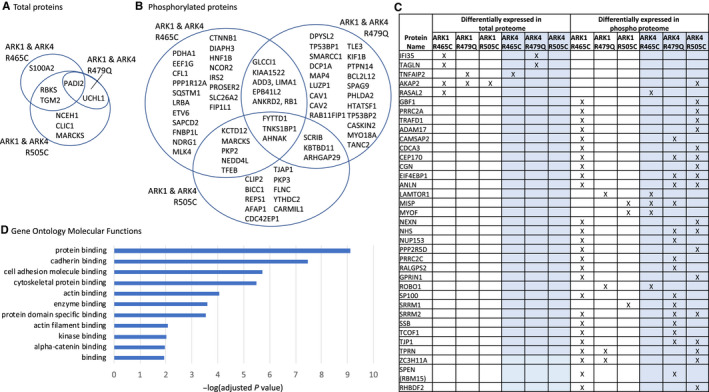
A total of 110 unique proteins were significantly (*P* < .05) differentially expressed in at least one ARK1 *and* one ARK4 CRISPR‐edited *FBXW7*‐mutant cell line compared to the matched parental cell line. Venn diagrams of total (A) and phosphorylated proteins (B) that were significantly (*P* < .05) differentially expressed in ARK1 and ARK4 CRISPR‐edited *FBXW7*‐mutated cell lines harboring the same *FBXW7* mutation, compared to matched parental cells. (C) Proteins not shown in (A) and (B) that were altered in *FBXW7*‐mutated cell lines harboring different mutations in ARK1 and ARK4 or in different proteomic analyses; “X” indicates that the protein was significantly differentially expressed in the *FBXW7*‐mutated cell line compared to the respective parental control. (D.) Significant Gene Ontology molecular functions resulting from g:Profiler^17^ (https://biit.cs.ut.ee/gprofiler/gost) analysis of 110 unique proteins differentially expressed in at least one ARK1 and ARK4 CRISPR‐edited *FBXW7‐*mutated cell line compared to matched parental cells (110 = combination of proteins listed in (A), (B), and (C); MARCKS is included in both (A) and (B))

**Figure 8:**
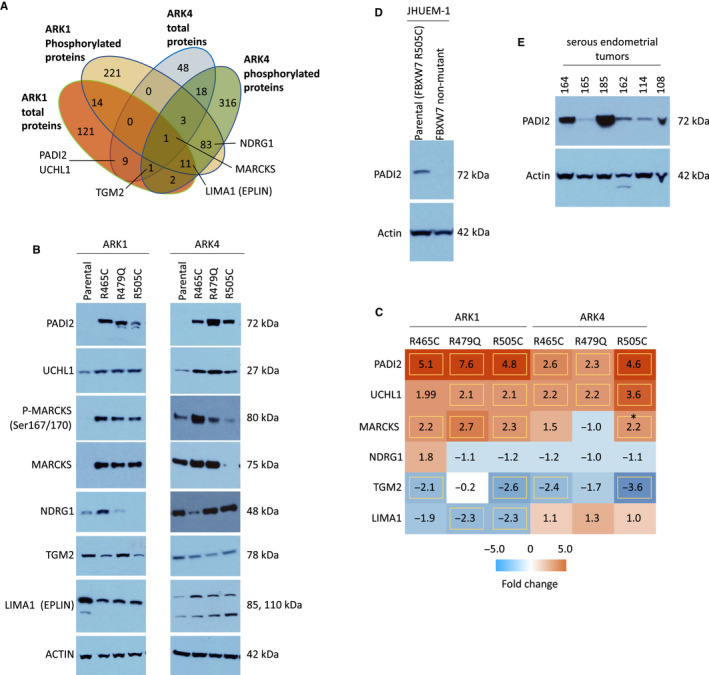
(A) Venn diagram of proteins significantly (*P* < .05) differentially expressed in CRISPR‐edited *FBXW7*‐mutated ARK1 and ARK4 cell lines compared to matched parental cells. Proteins validated via Western blot are labeled. (B) Western blot results confirming altered protein expression in ARK1 and ARK4 CRISPR‐edited *FBXW7*‐mutated lines compared to matched parental controls. All results are representative of a minimum of three lysate replicates from each cell line. (C) Intensity plot of average fold change (*FBXW7*‐mutated line compared to parental line) of total protein expression generated by mass spectrometry. Yellow boxes indicate values that met filtering criteria (≥±2.0 average fold change, *P* < .05 for difference between *FBXW7*‐mutated cell line compared to respective parental line). *Increased MARCKS total protein expression in ARK4 FBXW7 R505C cells compared to parental cells was only observed in one of three replicate Western blot experiments; blot shown is representative of the other two replicates. (D) CRISPR editing of JHUEM‐1 endometrial endometrioid adenocarcinoma cells (which endogenously harbor FBXW7 p.R505C) to a *FBXW7* nonmutant genotype resulted in decreased PADI2 expression. Results are representative of three lysate replicates. (E) Western blot detection of PADI2 protein expression in six serous endometrial tumors

We aimed to identify proteins that were changed in both biologically distinct serous EC cell lines (ARK1 and ARK4). A sum of 110 unique total and phosphorylated proteins were differentially expressed in at least one ARK1 *and* one ARK4 CRISPR‐edited *FBXW7*‐mutant cell line compared to the matched parental cell line (Figure 7A‐C). GO terms most significantly (*P* < 3.3 × 10^−6^) associated with these proteins were *protein binding*, *cadherin binding*, *cell adhesion molecule binding*, and *cytoskeletal protein binding* (Figure 7D, Table S17). Networks generated using Ingenuity Pathway Analysis indicated that these 110 proteins act in pathways known to contribute to serous ECs, including the mitogen‐activated protein kinase (MAPK), phosphatidylinositol‐4,5‐bisphosphate 3‐kinase (PI3K), and tumor protein 53 (p53) pathways (Figure S2). Of these 110 differentially expressed proteins, PADI2 was conspicuous in that it exhibited increased expression (2.3‐ to 7.6‐fold change, Figure 8C) across all *FBXW7*‐mutant lines compared to respective parental lines (Figure 7A) and is potentially directly druggable.

We used Western blot analyses to orthogonally validate the increased protein expression of PADI2 in all ARK1 and ARK4 *FBXW7*‐mutant cell lines as well as the expression changes of five additional proteins (UCHL1 (ubiquitin C‐terminal hydrolase L1), MARCKS (myristoylated alanine‐rich protein kinase C substrate), NDRG1 (N‐myc downstream regulated 1), TGM2 (transglutaminase 2), and LIMA1 (LIM domain and actin binding 1)) that were differentially expressed across different ARK1 and ARK4 derivative lines and for which antibodies were readily available (Figure 8A‐C). To further confirm the correlation between *FBXW7* mutation and PADI2 protein expression, we reverted the endogenous *FBXW7* c.C1513T (p.R505C) mutation to wild‐type in JHUEM‐1 endometrioid EC cells. Compared to parental cells, CRISPR‐edited *FBXW7* wild‐type JHUEM‐1 cells exhibited decreased PADI2 expression (Figure 8D), consistent with the increased PADI2 expression observed in ARK1 and ARK4 *FBXW7*‐mutant cells (Figure 8B). Finally, because PADI2 protein expression has not previously been shown in primary serous endometrial tumors, we demonstrated the expression of PADI2 in six primary tumors (Figure 8E, Table S18).

## 4 DISCUSSION

Serous ECs are challenging tumors associated with metastasis, recurrence, and poor outcome.^1^, ^2^ Research over the last decade has revealed molecular aberrations that occur in these aggressive tumors and ongoing efforts are aimed at determining the functional consequences and clinical utility of these abnormalities. Given the high frequency of somatic *FBXW7* mutation in serous ECs (range 15%‐29%; reviewed in Ref. [^3^]), we sought to identify potentially druggable protein changes associated with three recurrent somatic *FBXW7* mutations (p.R465C, p.R479Q, and p.R505C), which account for 34% of all *FBXW7* mutations reported in serous endometrial tumors.^5^, ^6^, ^19^

Through LC‐MS/MS‐based analyses and Western blot validation, we identified upregulated PADI2 protein in conjunction with all *FBXW7* hotspot mutations. To our knowledge, this is the first report of PADI2 protein expression in serous ECs and the first association between increased PADI2 protein and *FBXW7* mutations in any tumor type. Furthermore, our correlation of increased PADI2 expression to an endogenous *FBXW7* mutation (p.R505C) in JHUEM‐1, a grade 2, microsatellite instable (inferred by Ref. [^10^]) endometrioid EC cell line, indicates that our results may be generalizable across EC histological and molecular subtypes.

Herein, we demonstrated PADI2 protein expression in six primary endometrial tumors but lacked appropriate matched normal endometrium samples for comparison. However, the expression of PADI2 has been characterized in non‐uterine cancers. Compared to normal tissue, PADI2 protein is increased in breast, cervical, colon, liver, lung, ovarian serous, thyroid,^20^ and prostate cancers.^21^ PADI2 mRNA expression is upregulated in tamoxifen‐resistant^22^ and HER2/ERBB2 + breast cancers.^23^ In contrast, in colon cancer, PADI2 mRNA and protein are decreased^24^, ^25^ and low mRNA correlates with poor prognosis.^24^

PADI2 catalyzes citrullination, whereby peptidylarginine is converted into peptidyl‐citrulline. Substrates of PADI2 include histones, vimentin, β‐ and γ‐actins, myelin basic protein,^26^ and β‐catenin.^27^ In preclinical models of breast cancer,^28^, ^29^, ^30^, ^31^, ^32^ prostate cancer,^21^, ^33^ gastric cancer,^20^ and glioblastoma,^34^ PADI2 inhibition or depletion decreased growth, migration, and extracellular vesicle release, and was synergistic with docetaxel and bortezomib in inducing apoptosis in tamoxifen‐resistant breast cancer ^22^ and bone marrow mesenchymal stem cells,^35^ respectively. Additionally, overexpression of PADI2 in a transgenic mouse model caused the development of spontaneous skin neoplasms.^36^ In contrast, in colon cancer cells, overexpression of PADI2 decreased growth^25^, ^27^ while deletion of PADI2 increased growth, reduced cell contact inhibition, and reduced sensitivity to nitazoxanide.^27^ We anticipate future research into the functional effects of PADI2 dysregulation in ECs.

Our results provided insight into the global proteomic changes associated with *FBXW7* mutations in serous EC cells. Our stringent filtering criteria led to the identification of proteins that were reproducibly altered across triplicate lysates (Figures 2 and 5) and included 21 proteins identified as known or potential FBXW7 substrates differentially expressed in HCT116 *FBXW7* knockout colorectal cancer cells compared to wild‐type cells^37^ (Table S19). Among these proteins was cyclin E1, which validates a previous report of increased cyclin E1 in *FBXW7*‐mutant serous EC cells.^7^ We also orthogonally validated six additional proteins that were differentially expressed in ARK1 and ARK4 *FBXW7*‐mutant cells (Figure 8B). Two of these proteins, PADI2 (described above) and UCHL1, are potentially directly druggable^38^, ^39^ and exhibited increased total protein expression in all *FBXW7*‐mutant derivative cell lines across two genomically distinct backgrounds. Interestingly, UCHL1 promotes p53 signaling in nasopharyngeal carcinoma^40^ and PADI2 facilitates p53 degradation in breast cancer cells.^22^ This raises the possibility that the increase in PADI2 we observe is a response to increased UCHL1 protein expression, or vice versa. While our research was ongoing, increased UCHL1 RNA expression was shown to be prognostic for poor overall survival for serous EC patients,^41^ a result previously shown in a cohort of mostly endometrioid EC patients.^42^ Immunohistochemical expression of UCHL1 in serous ECs and reduced proliferation and cell cycle progression with UCHL1 silencing were also published in this same report.^41^ Our results provide the additional novel insight that UCHL1 expression is affected by *FBXW7* mutation.

The data we provide herein is a valuable compendium of protein expression altered by *FBXW7* mutation but has several limitations. For example, a direct comparison of phosphorylated proteins and total proteins identified in this study is difficult because we utilized label‐free IMAC enrichment for phosphorylated peptides and TMT tags to enrich total proteins; however, the overlap of significantly differentially expressed proteins identified by both methods ranged from 5 to 18 proteins (Table S20). It is also unknown whether the proteomic changes we identified would occur with alternate *FBXW7* mutations, *FBXW7* copy number alterations, or in other genomic backgrounds. Future determination of whether PADI2 expression correlates with *FBXW7* mutation in uterine carcinosarcomas, which have somatic *FBXW7* mutation rates of 11%‐39% (reviewed in Ref. [3]), will be important. Finally, our work was restricted to the identification of proteins altered by *FBXW7* mutation; however, the results provided herein combined with recent literature implicating the function of PADI2 in other tumor types and of UCHL1 in serous ECs, as well as the potential to directly drug both proteins provides significant impetus for future research.

CRISPR‐edited ARK1^7^ and ARK4 *FBXW7*‐mutant derivative serous EC cell lines are important resources to aid the study of *FBXW7* mutations in this rare tumor type. By comparing the proteomic profiles of *FBXW7*‐mutant derivative lines to matched parental lines, we identified changes that occur across cells harboring distinct *FBXW7* mutations (p.R465C, p.R479Q, and p.R505C) and across cells with different genomic backgrounds (ARK1 and ARK4). We orthogonally validated expression changes in a number of proteins, including increased PADI2 in all *FBXW7*‐mutant cell lines. We further confirmed increased PADI2 protein in FBXW7 p.R505C‐mutant JHUEM‐1 endometrioid EC cells compared to CRISPR‐edited *FBXW7* nonmutant JHUEM‐1 cells. Finally, we are the first to demonstrate PADI2 protein expression in primary serous endometrial tumors. These results lay a solid foundation for future research to investigate the therapeutic potential of PADI2 in serous and other *FBXW7*‐mutated ECs.

## CONFLICT OF INTEREST

Mary Ellen Urick has no conflict of interest. In the interest of full disclosure, Daphne W. Bell receives royalty income from US patent No. 7,294,468 for work unrelated to the current study.

## AUTHOR CONTRIBUTIONS

Daphne W. Bell co‐conceptualized and supervised the study and provided critical edits to the article. Mary Ellen Urick co‐conceptualized the study, performed all experiments not designated to a contracting company in the methods, completed formal analysis, and data curation, and wrote the original draft of the article.

## Supporting information

Fig S1‐S2

Table S1‐S8

Table S9‐S20

## Data Availability

Additional data supporting our study conclusion are presented as supplementary material and are available from the corresponding author upon reasonable request. The data are not publicly available due to privacy and ethical restrictions.
