# Supplementary material for: Proteomic profiling of FBXW7‐mutant serous endometrial cancer cells reveals upregulation of PADI2, a potential therapeutic target
Source: Cancer Med. 2020 Apr 5;9(11):3863–74. doi: 10.1002/cam4.3013 (PMC7286459; doi:10.1002/cam4.3013)

Supplemental Figure 1

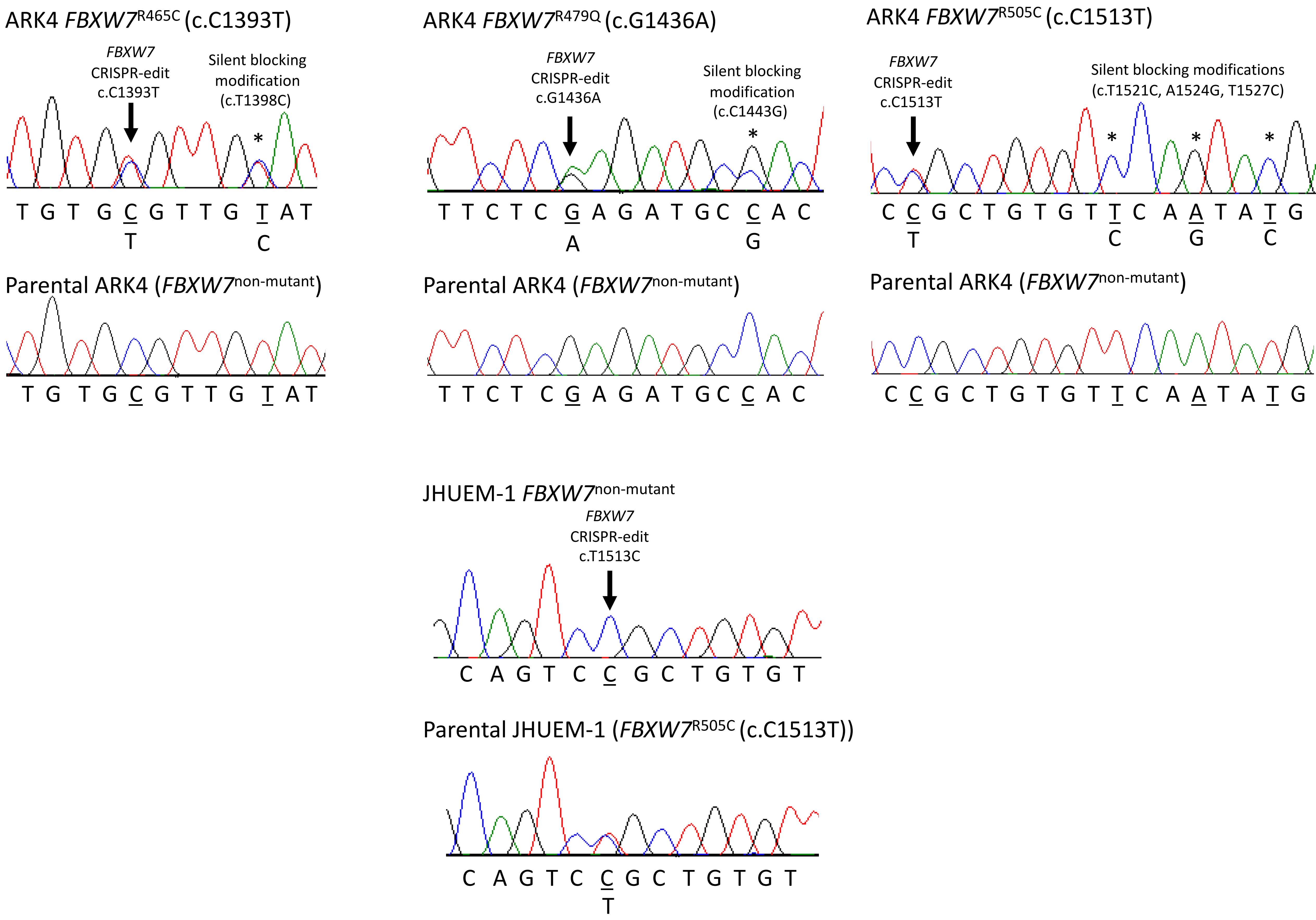

Supplemental Figure 2

A.

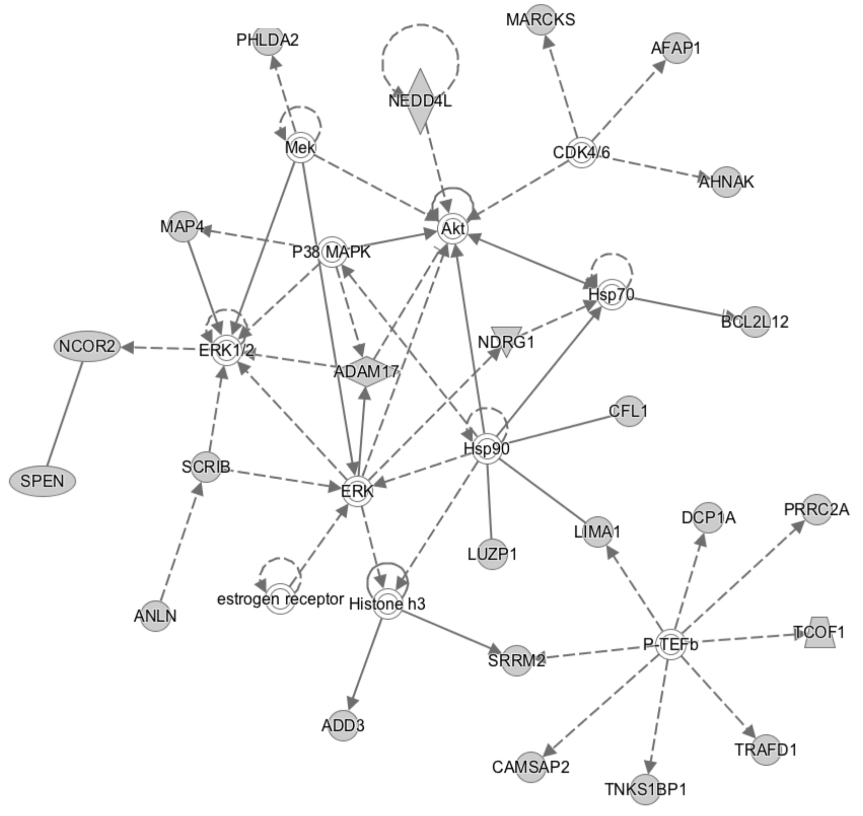

B.

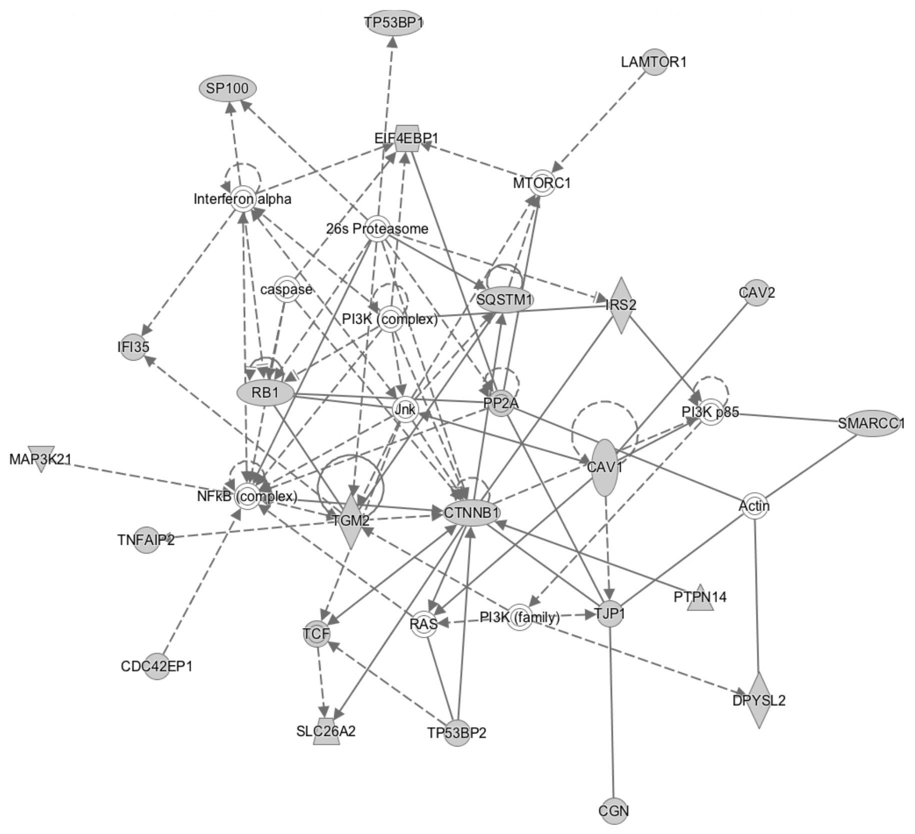

C.

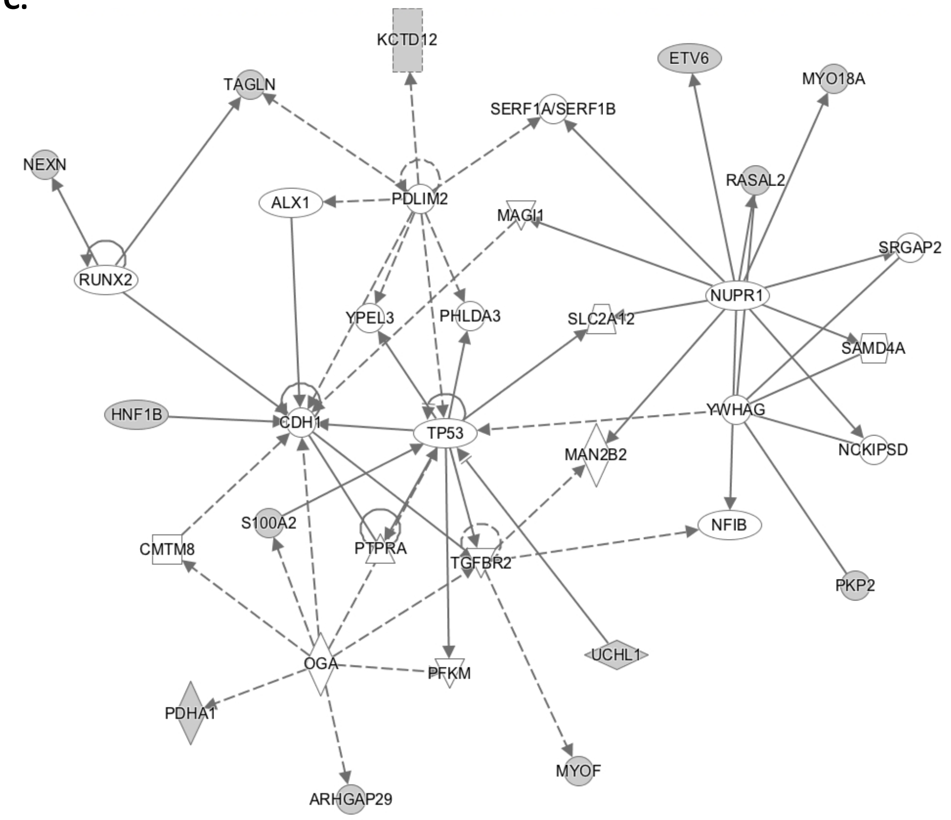

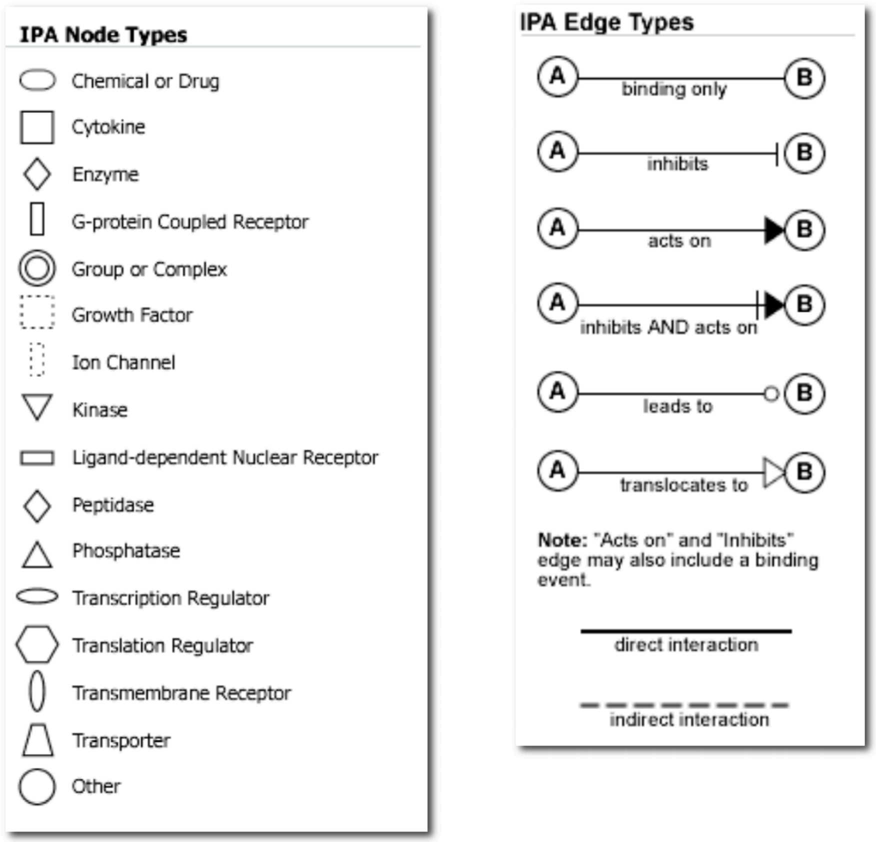

Supplement: Supplementary file 1 — Fig S1‐S2 [file CAM4-9-3863-s001.pdf]
